# Failure of *Staphylococcus aureus* to Acquire Direct and Cross Tolerance after Habituation to Cinnamon Essential Oil

**DOI:** 10.3390/microorganisms7010018

**Published:** 2019-01-11

**Authors:** Xiaoqiu Song, Yue Sun, Qian Zhang, Xiaobo Yang, Feng Zheng, Shoukui He, Yifei Wang

**Affiliations:** School of Perfume and Aroma Technology, Shanghai Institute of Technology, Haiquan Road 100, Shanghai 201418, China; Songxiaoqiu@sit.edu.cn (X.S.); 13685413920@163.com (Y.S.); Zqq_951208@163.com (Q.Z.); y8621@163.com (X.Y.); andy4zf@163.com (F.Z.)

**Keywords:** essential oil, stress tolerance, *Staphylococcus*, stress adaptation

## Abstract

Utilization of sublethal concentrations of cinnamon essential oil (CEO) for food preservation has been proposed. However, exposure to stressful, sublethal growth conditions may induce bacterial tolerance to homologous or heterologous stressing agents. Hence, the ability of CEO to stimulate bacterial stress response was evaluated in the current work. *Staphylococcus aureus* was exposed to 1/4 and 1/2 of the minimum inhibitory concentration (MIC, 500 μL/L) of CEO for 18 h. It was found that overnight habituation to CEO failed to induce direct tolerance and cross-tolerance to lactic acid (pH 4.5), NaCl (10 g/100 mL) and high temperature (45 °C) in *S. aureus*. Likewise, *S. aureus* cells subjected to successive habituation with increasing amounts (1/16 MIC to 2× MIC) of CEO developed no direct tolerance. Taken together, CEO has no inductive effect on the acquisition of stress tolerance in *S. aureus*.

## 1. Introduction

*Staphylococcus aureus* is a common cause for foodborne disease due to the capacity of certain strains to produce staphylococcal enterotoxins [1]. *S. aureus* is also notorious for its ability to develop resistance to antibiotics [2]. Moreover, this bacterium is able to mount tolerance to salt, heat and acid stresses when exposed to sublethal environmental conditions [3,4]. The acquired tolerance to food processing-related stresses in *S. aureus* has motivated the development of novel strategies to control this organism in foods.

Essential oils (EOs) have received worldwide attention for their remarkable antimicrobial capacity. The potential of EOs to extend the shelf life of a wide variety of foods (e.g., juices, meat and meat products) has been demonstrated [5,6]. In our previous studies, cinnamon essential oil (CEO) stood out for its antibacterial efficacy against *S. aureus* [7,8]. Furthermore, CEO exhibited a satisfactory antimicrobial performance on methicillin-resistant *S. aureus* and its biofilm [9]. However, the application of EOs is partially limited by their intense aroma. The amounts of EOs required to inhibit bacteria in situ are frequently organoleptically unacceptable [10,11]. A proposed approach is to combine sublethal concentrations of EOs with other preservation methods [12]. Nevertheless, habituation to a sublethal stress can confer bacteria tolerance to the same type of stress (direct tolerance) or seemingly unrelated stresses (cross tolerance) [13,14].

Bacterial stress response to EOs has received much attention in recent years. The main reason for this investigation is to elucidate whether alteration in bacterial tolerance is a major issue when considering the use of EOs as a potential antimicrobial in food systems. Gomes-Neto et al. (2012a, 2012b, 2012c, 2014) found that *Rosmarinus officinalis* L. EO did not induce direct or cross tolerance in *Pseudomonas aeruginosa*, *Listeria monocytogenes* and *Salmonella typhimurium* [15,16,17,18]. Furthermore, exposure of the above-mentioned pathogens and *S. aureus* to *Origanum vulgare* L. EO revealed no development of stress tolerance [19,20,21,22,23]. Despite the strong antibacterial activity of CEO, there is a lack of information regarding the ability of bacteria to develop stress tolerance following exposure to sublethal amounts of CEO. Hence, the current study aimed to examine whether CEO habituation induces direct and cross tolerance in bacteria, employing *S. aureus* as a model organism.

## 2. Materials and Methods

### 2.1. Essential Oil

Food-grade CEO, purchased from Hui County Fragrant Plant Development Co., Ltd. (Gansu, China), was stored at 4 °C in brown glass bottles. Its major components were trans-cinnamaldehyde (86.07%), trans-cinnamic acid (11.04%) and benzaldehyde (1.28%) as revealed by gas chromatography-mass spectrometer analysis in our previous study [24].

### 2.2. Target Bacterium

*S. aureus* CICC 23478 was provided by Center of Industrial Culture Collection, Beijing, China. A stock culture was maintained on nutrient agar (NA) (Beijing Land Bridge Technology Co. Ltd., Beijing, China) at 4 °C. Prior to each experiment, a single colony from NA was inoculated into Luria-Bertani (LB) broth, followed by incubation at 37 °C/200 rpm for 18 h. The cells were harvested by centrifugation, washed with sterile distilled water and suspended in sterile normal saline (0.85%) to a concentration of appropriately 8 log_10_ CFU/mL.

### 2.3. Determination of Minimum Inhibitory Concentration (MIC)

The MIC of CEO against *S. aureus* was determined by two-fold serial dilution method [14,25]. A series of two-fold dilutions of CEO were prepared with 100% ethanol to facilitate dissolution. An aliquot of 50 μL CEO dilutions was added to 5 mL LB broth, achieving a final concentration ranging from 4000 to 62.5 μL/L. The same amount of ethanol and sterile distilled water were added as controls. To each tube, 50 μL of bacterial suspension was inoculated so that the final concentration of viable bacteria was about 6 log_10_ CFU/mL. The final concentration of ethanol in the tubes was from 6 to 10 μL/mL, which did not inhibit the growth of *S. aureus* as demonstrated in our preliminary test. Bacterial growth was monitored by measuring the absorbance at 600 nm after incubation at 37 °C for 24 h. The lowest concentration of CEO, resulting in complete inhibition of visible growth of *S. aureus*, was determined as MIC.

### 2.4. Induction of Bacterial Direct and Cross Tolerance

The induction of direct and cross tolerance in *S. aureus* was carried out according to Luz et al. (2012a) [19]. Briefly, an aliquot of 50 μL bacterial suspension was inoculated into 5 mL of LB broth containing 1/4 MIC and 1/2 MIC of CEO, followed by incubation at 37 °C for 18 h to prepare the CEO-habituated cultures. The non-habituated control was prepared similarly without CEO. Afterwards, 50 μL of CEO-habituated and non-habituated cultures were added to 5 mL of LB broth containing CEO at its MIC value. The viable population of *S. aureus* was determined after incubation at 37 °C for 0, 1, 2 and 3 h. Ten-fold serial dilutions of samples were prepared with sterile normal saline (0.85%). Then, a 100 μL aliquot of appropriate dilutions was plated on NA and incubated at 37 °C for 24 h. Colonies were counted and expressed as log_10_ CFU/mL.

In a cross tolerance test, the susceptibility of *S. aureus* to salt (10% NaCl), lactic acid (pH 4.5) and heat (45 °C) stresses was determined. The criteria for the selection of these conditions were: (i) the combination of mild control measures (also referred to as hurdle technology) has been proposed for food preservation [26]; and (ii) these stressors were modestly inhibitory against the target bacterium. *S. aureus* was habituated to CEO as described above. An aliquot of 50 μL CEO-habituated and non-habituated cultures was inoculated into 5 mL of LB broth containing 10% NaCl or adjusted to pH 4.5 by lactic acid to evaluate salt and acid tolerance, followed by incubation at 37 °C. In a heat tolerance test, a 50 μL culture from each case was added to 5 mL LB of broth and incubated at 45 °C. The viable population of *S. aureus* was determined after incubation for 0, 1, 2 and 3 h.

The capacity of *S. aureus* to develop direct tolerance was also determined through successive 24-h habituation to increasing amounts (1/16 MIC, 1/8 MIC, 1/4 MIC, 1/2 MIC, MIC and 2× MIC) of CEO according to To et al. (2002) with modifications [27]. Briefly, 50 μL of bacterial suspension was inoculated into 5 mL of LB broth containing 1/16 MIC of CEO and incubated at 37 °C for 24 h. The viability of *S. aureus* was determined at the end of incubation. Simultaneously, 50 μL of the above-mentioned overnight culture was inoculated into 5 mL of fresh LB broth containing a higher amount of CEO (1/8 MIC). Then, the tube was incubated overnight at 37 °C and the cell number was detected similarly. This cycle was repeated by gradually increasing the concentration of CEO until no colony information was observed. Otherwise, the CEO concentration was increased to 2× MIC.

### 2.5. Statistical Analysis

All the experiments were carried out in triplicate on two separate occasions. Data were subjected to a one-way ANOVA analysis using statistical software SAS v. 8.0 (SAS Institute, Cary, NC, USA). Turkey’s HSD test was employed to detect the differences in population between CEO-habituated and non-habituated *S. aureus*. Difference at the *p* < 0.05 level was considered to be significant.

## 3. Results and Discussion

### 3.1. CEO Exhibited Antimicrobial Activity against S. aureus

The capacity of *S. aureus* to acquire tolerance to many antimicrobial procedures employed by the food industry to ensure food safety has motivated the research into natural EOs to control this bacterium in foods [23]. CEO has been confirmed as a strong antimicrobial agent due to its ability to disrupt bacterial cell membrane and metabolic activity [28]. In the current study, CEO exhibited a MIC value of 500 μL/L against *S. aureus*. In contrast, Lu et al. (2011) reported that the MIC of CEO against *S. aureus* was 400 μL/L [29]. Variations in the test microorganism, composition of CEO and some other intrinsic and extrinsic factors can explain the differences in MIC of CEO [28]. Furthermore, 1/2 MIC and 1/4 MIC of EOs have demonstrated an inhibitory but not lethal effect on bacteria [17]. Hence, these concentrations of CEO were utilized in subsequent tests to prepare CEO-habituated cells of *S. aureus*.

### 3.2. Overnight Exposure of S. aureus to CEO Did Not Induce Direct and Cross Tolerance

**Figure 1:**
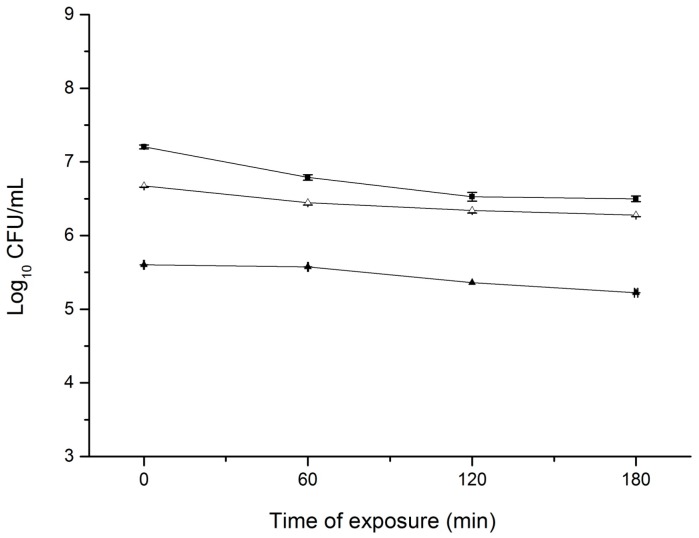
Survival of non-habituated and cinnamon essential oil (CEO)-habituated *S. aureus* in Luria-Bertani (LB) broth containing CEO at its minimum inhibitory concentration (MIC) level (500 μL/L). (■) non-habituated cells; (Δ) cells pre-habituated with 1/4 MIC (125 μL/L) of CEO; (▲) cells pre-habituated with 1/2 MIC (250 μL/L) of CEO. Vertical bars represent standard deviation. Data point symbols without visible error bars signify the standard deviation smaller than the symbol size.

Overnight habituation in sublethal amounts (1/2 MIC and 1/4 MIC) of CEO did not induce bacterial direct tolerance as demonstrated by the viable cell counts over 180 min (Figure 1). The survival behavior of non-habituated and CEO-habituated *S. aureus* was similar when further cultivated in LB broth containing the MIC level of CEO. A slight drop (≤0.7 log_10_ CFU/mL) in population was observed for all three groups after incubation for 180 min. However, there was no marked difference in population reduction between non-habituated and CEO-habituated cultures. A similar result was obtained by Luz et al. (2012b) when evaluating the development of direct tolerance in *L. monocytogenes* after adaptation to carvacrol (the major component of *Origanum vulgare* L. EO) [20].

The ability of *S. aureus* to acquire direct tolerance depends largely on *de novo* protein synthesis. For example, the addition of protein synthesis inhibitors (e.g., rifampicin and chloramphenicol) to the adaptation medium completely abolished the development of homologous tolerance to acid and hydrogen peroxide in *S. aureus* [4]. Cinnamic acid, a major component of CEO, was found to inhibit *S. aureus* enzymes related to ATP production and glucose uptake [30]. ATP synthesis is necessary for membrane potential, pigmentation and cell wall synthesis to form colonies [31]. Furthermore, sublethal concentrations of CEO decreased the expression of genes encoding enterotoxins A, C and E as well as cell division protein FtsZ in *S. aureus* [30,32]. Suppression of FtsZ function can lead to bacterial filamentation and cell lysis [30]. These findings suggest a correlation between lack of direct tolerance induction and inhibition of protein synthesis by CEO.

**Figure 2:**
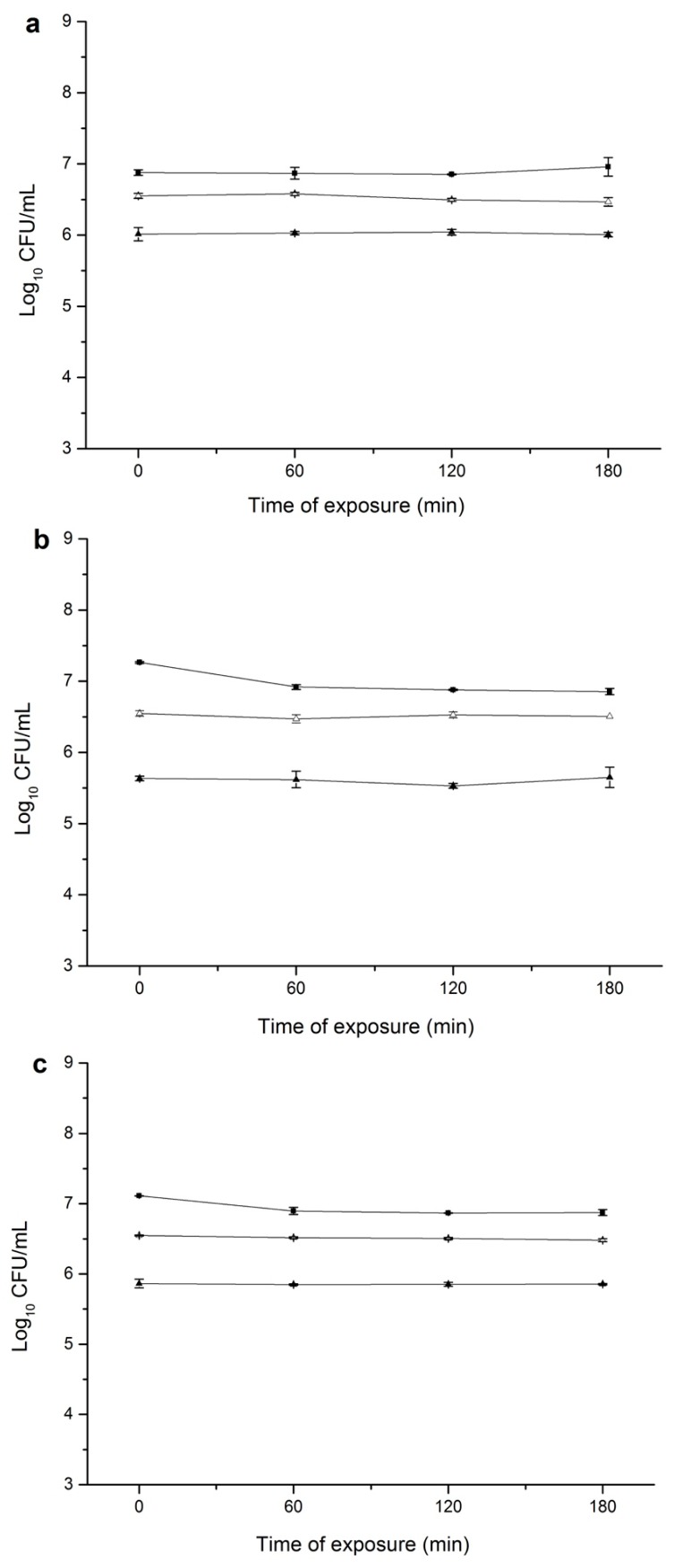
Survival of non-habituated and CEO-habituated *S. aureus* in LB broth at 45 °C (**a**); with 10 g/100 mL NaCl (**b**); or acidified with lactic acid to pH 4.5 (**c**). (■) non-habituated cells; (△) cells pre-habituated with 1/4 MIC (125 μL/L) of CEO; (▲) cells pre-habituated with 1/2 MIC (250 μL/L) of CEO. CEO, cinnamon essential oil; MIC, minimum inhibitory concentration. Vertical bars represent standard deviation. Data point symbols without visible error bars signify the standard deviation smaller than the symbol size.

In agreement with the findings from direct tolerance test, *S. aureus* cells submitted to CEO habituation exhibited no acquisition of cross-tolerance to salt, lactic acid and high temperature as determined by the viable cell counts (Figure 2). CEO-habituated and non-habituated *S. aureus* displayed a quite similar profile when further cultivated under these heterologous stressing conditions. The cell number for both cultures was generally maintained throughout the 180 min of exposure. Similarly, *Rosmarinus officinalis* L. EO and *Origanum vulgare* L. EO did not confer cross tolerance to several foodborne pathogens including *L. monocytogenes*, *S. typhimurium* and *P. aeruginosa* [15,16,17,18,19,20,21,22,23].

The induction of bacterial cross-tolerance responses between different stresses can be attributed to the overlapping roles of some shock proteins [33]. The synthesis of heat shock proteins and acid shock proteins is necessary for the development of tolerance to heat and acid in *S. aureus*, respectively [4]. Moreover, accumulation of compatible solutes (e.g., glycine betaine, choline and proline) contributes to the survival of this bacterium under osmotic stress [34]. Hence, failure of *S. aureus* to mount cross tolerance in the current work may be because this bacterium does not use heat, acid or osmotic stress tolerance networks to manage CEO habituation.

### 3.3. Repeated Exposure of S. aureus to Increasing Amounts of CEO Did Not Induce Direct Tolerance

The induction of direct tolerance was also explored by continuous exposure to increasing concentrations of CEO (1/16 MIC to 2× MIC) through successive 24 h cycles. It was found that *S. aureus* was able to survive in LB broth containing up to the MIC level of CEO. This result suggests that repeated habituation to CEO for a more prolonged time did not stimulate significant changes in bacterial susceptibility to CEO when assessed by the standard MIC evaluation criteria [35]. Likewise, *S. typhimurium* progressively exposed to increasing amounts of *Origanum vulgare* L. EO was able to survive up to the MIC of this oil [19]. Our previous work demonstrated that CEO at MIC and minimum bactericidal concentration (MBC) levels decreased the membrane potential (MP) of *S. aureus* [8]. MP plays a crucial role in ATP generation and bacterial physiology. Disturbance of MP can depolarize the cell membrane, thus resulting in disruption of metabolic activity and bacterial growth [8]. This may be a reason for the failure of *S. aureus* to develop direct tolerance after repeated exposure to CEO in the current work.

It should be noted that some authors isolated stress-tolerant strains by parallel evolution [36,37]. For instance, Horinouchi et al. (2010) obtained six ethanol-tolerant mutants after 1000-generation cultivation in 5% ethanol, an antibacterial agent targeting cell membranes as EOs do [37]. It would be meaningful to explore if CEO-insusceptible mutants can occur in this way in future investigations. On the other hand, the current work employed a strain of *S. aureus* to characterize bacterial stress response to CEO. Additionally, a single bacterium was utilized as a model organism in many previous studies to assess the development of bacterial stress tolerance following habituation to EOs [15,16,17,18,19,20,21,22]. Hence, a population-wide survey on the diversity in bacterial response to EOs merits further investigation.

Some attention has been paid to stress tolerance of *S. aureus* after habituation to EOs in recent years. Thomsen et al. (2013) found that exposure of *S. aureus* to tea tree (*Melaleuca alternifolia*) EO did not stimulate tolerance to itself or to other antimicrobials (e.g., carvacrol, triclosan, terpinen-4-ol, chloramphenicol, fusidic acid, linezolid and mupirocin) [38]. Moreover, *Origanum vulgare* L. EO failed to induce direct tolerance and cross-tolerance to salt, organic acid and high temperature in *S. aureus* [21,23]. Similarly, no induction of stress tolerance was observed in *S. aureus* following short or long-term exposure to CEO in the current work. These findings are quite interesting in light of previously reported development of acid, heat and hydrogen peroxide tolerance induced by sublethal stress conditions in *S. aureus* [4].

## 4. Conclusions

This is the first report assessing the ability of a bacterium to develop stress tolerance after exposure to sublethal amounts of CEO in LB broth. The results revealed that CEO failed to induce direct tolerance and cross-tolerance to NaCl, lactic acid and high temperature in *S. aureus*. Overall, this work suggests that alteration in bacterial tolerance is not a major issue when considering the utilization of CEO as an anti-*S. aureus* agent. Investigations regarding CEO habituation of *S. aureus* in a meat-based or vegetable-based broth are underway to further support this statement.
